# Gender, perceived competence and the enjoyment of physical education in children: a longitudinal examination

**DOI:** 10.1186/1479-5868-9-26

**Published:** 2012-03-06

**Authors:** John Cairney, Matthew YW Kwan, Scott Velduizen, John Hay, Steven R Bray, Brent E Faught

**Affiliations:** 1 Department of Family Medicine, McMaster University, 175 Longwood Road South, Suite 201A, Hamilton, ON L8P 0A1, Canada; 2 Centre for Addiction & Mental Health, 33 Russell St, Toronto, ON M5S 2S1, Canada; 3 Department of Community Health Sciences, Brock University, 500 Glenridge Ave, St., Catharines, ON L2S 3A1, Canada; 4 Department of Kinesiology, McMaster University, 1280 Main Street West, Hamilton, ON L8S4L8, USA

**Keywords:** Prospective cohort study, Athletic competence, Physical education

## Abstract

**Background:**

The current study examined associations between gender, perceived athletic competence, and enjoyment of physical education (PE) class over time in a cohort of children enrolled in grade four (ages 9 or 10) at baseline (n = 2262).

**Methods:**

We assessed each student 5 times over a period of 2 years. We used mixed effects modeling to examine change over time in enjoyment of PE.

**Results:**

Enjoyment of PE declined among girls but remained constant among boys. Higher levels of perceived competence were associated with higher PE enjoyment. A 3-way interaction between gender, competence, and time revealed that PE enjoyment was lowest and declined most markedly among girls with low perceived athletic competence. Among boys with low competence, enjoyment remained at a consistently low level.

**Conclusions:**

Our results indicate that lower perceived athletic competence is associated with low enjoyment of PE, and, among girls, with declining enjoyment. Findings suggest that interventions in a PE context that target perceived competence should be considered in future work.

## Introduction

Regular engagement in physical activity is associated with a variety of psychological, social and physical benefits for children and youth [1,2]. Unfortunately, previous research has consistently demonstrated that physical activity decreases during the transition from childhood to adolescence, with girls in particular showing significant declines in participation [3-5]. There is no widely accepted explanation for this phenomenon; however, several authors have suggested that negative experiences in, and perceptions of, school-based physical education (PE) class may be an important factor in the decline in physical activity, particularly among girls [6]. This decline occurs at the same time PE becomes an optional part of the curriculum in most school systems in North America [7].

School-based PE programs are often touted as an effective means of positively affecting health and physical activity behaviours during childhood [2] and later life [8]. School programs reach the vast majority of children [9], and can affect physical activity both directly and indirectly. PE classes can provide children with activity that directly fosters motor skill development and fitness, and can also stimulate positive perceptions of physical activity - thereby influencing motivation to engage in physical activity [6-10]. Unfortunately, declining physical activity participation during adolescence suggests that there are deficiencies in the current PE programs being implemented. Moreover, it remains unclear whether students' experiences, or perceptions of those experiences, in school PE truly facilitate physical activity over the long term.

One important determinant of physical activity is simply youths' perceived enjoyment of such activity. Data from several studies show that motivation to participate in physical activity in children is influenced by perceptions of physical activity as fun, interesting, and challenging [11,12]. Enjoyment of physical activity is positively correlated with physical activity participation levels for children and adolescents [13-16]. Enjoyment of PE class specifically has also been identified as a predictor of physical activity [17]. Sallis and colleagues [14] investigated 22 potential correlates of physical activity in school children and found that students' enjoyment of PE class was a robust predictor of physical activity at both younger (grades 4-6) and older (grades 7-12) ages. Given its strong link to physical activity in children and youth, further investigation of enjoyment within the context of PE classes is warranted.

Numerous theories of motivated behavior that have been applied to understanding and predicting physical activity participation (e.g., social cognitive theory, competence motivation theory, self-determination theory) highlight positive affect (e.g., fun, enjoyment) as a proximal motivator of physical activity. Positive affect, in turn, is influenced by more distal perceptions such as self-efficacy or competence, outcome expectations, autonomy, and relatedness. In the literature on children's physical activity, perceptions of competence, autonomy, and perceived success have been shown to predict physical activity enjoyment, as well as to reduce negative perceptions, such as boredom [18,19]. In the school environment, self-efficacy with respect to leisure-time physical activity was the strongest predictor of PE class enjoyment in a sample of middle school girls [6]. Together, these findings suggest that perceived competence may be an important factor affecting students' enjoyment of their experiences in PE.

The objective of the present study was to investigate associations between perceived athletic competence, PE class enjoyment, and gender in a sample of school-aged children across grades 4 to 6. We conducted a 3-year prospective study, allowing us to extend existing research in this field, which, with one exception [9], has relied exclusively on cross-sectional data [6,15,19-21]. Importantly, we were able to examine trends in perceptions of athletic competence and PE enjoyment over time. Based on cognitive theories of motivation and previous research in children's physical activity [e.g., 6-12], we hypothesized that perceived athletic competence would be a significant predictor of children's PE enjoyment.

Although the major objective of the study was to investigate the relationship between perceived competence and enjoyment of PE, we also set out to extend previous work investigating gender differences as they relate to PE enjoyment. In previous studies, girls have been found to enjoy PE less than boys [9,21,22]. It is possible that determinants of PE enjoyment vary by gender; however, our understanding of *why *girls differ from boys in their enjoyment of PE remains limited, primarily because studies have either simply established the presence of gender differences with no further elaboration or test of possible mechanisms [9], or have limited their sample to girls [6]. For example, the study by Barr-Anderson and colleagues [6] identified a number of individual and contextual factors associated with PE enjoyment; but, by excluding boys, the design precluded an examination of the sources of gender differences. In line with previous research, we hypothesized that girls would report lower PE enjoyment than boys. As an exploratory step, gender was examined as a potential moderator of the relationship between perceived athletic competence and PE enjoyment over time.

## Methods

### Study sample and data collection

The data for this study come from the Physical Health and Activity Study Team (PHAST), a longitudinal cohort study of the health, physical activity, and development of children. The target population for this study included all children enrolled in Grade 4 in the public school system of a large region in southern Ontario, Canada. In Year One (2004-05) of the study, we obtained ethics approval from the regional district school board and from Brock University. We received permission from 75 of 92 possible school principals (83%) and informed consent from the parents of 2262 of 2378 children (95.4%) in these schools. We established testing and training protocols, and completed pilot testing in the fall of 2004. Data collection began in May 2005, with follow-ups in the September 2005, May and September 2006, and May of 2007. We have data, therefore, on a cohort of children from the end of grade 4 through to the end of grade 6 (5 time points over three years). Sample sizes and descriptive statistics for key measures for each wave are shown in Table 1.

**Table 1:** Participant demographics over time

	Grade 4	Grade	5	Grade	6
	**Spring '05**	**Fall '05**	**Spring '06**	**Fall '06**	**Spring '07**

Sample Size (N)	2150	2103	2067	2030	1989

Enjoyment of Physical Education	10.8 (1.8)	10.8 (1.8)	10.8 (1.7)	10.8 (1.7)	10.8 (1.7)

Organized Activity Participation	5.8 (5.0)	4.6 (4.6)	5.8 (5.2)	4.2 (4.3)	5.6 (5.1)

Perceived Athletic Competence	18.4 (4.1)	18.6 (4.1)	18.9 (4.0)	18.9 (4.0)	19.1 (3.9)

Body mass index (kg/m^2^)	18.6 (3.5)	19.0 (3.8)	19.3 (3.9)	19.7 (4.0)	20.1 (4.0)

Mean scores and standard deviations (in parentheses) are reported

Teams of three to four trained research assistants tested children during regular school hours. Only those who demonstrated competence in the administration of all measurements, as assessed by the core research team, were allowed to take part in the study. Research assistants were randomly observed by the core research team to ensure that anthropometric procedures were being followed. Questionnaires were administered in the classroom under supervision of the team. On completion, team members escorted children to the gymnasium, where fitness and anthropometric assessments were completed. Steps were taken to ensure privacy during assessment of weight. All equipment was calibrated daily.

### Measures

#### Enjoyment of physical education

The dependent variable is a measure of perceived enjoyment of PE class [23]. Unlike previous work [6-9], we used a multi-item measure of perceived enjoyment of PE. Each item requires the child to decide which of two descriptions best describes him or her. This is accomplished by selecting one of a pair of sentences that present mutually exclusive descriptions. For example, "*Some children have fun in physical education class*", and "*Other kids would rather miss physical education class*". The child then indicates whether the sentence he or she selected was "*sort of true for me*" or "*really true for me*". There were three items tapping into enjoyment, fun, and perceived difficulty of games in PE. The enjoyment of PE class scale has good to excellent test-retest reliability (*r *= 0.70-0.89) as well as strong predictive and construct validity for children and adolescents 9 to 16 years of age [23]. Higher scores indicate greater enjoyment of PE class.

#### Perceived athletic competence

We used the Perceived Athletic Competence (PAC) subscale of the Harter Self-Perception Profile for Children (SPPC) [24] to measure children's views about their abilities with respect to physical activity. The SPPC employs a structured alternative choice format. Children are asked to choose one of a pair of sentences (e.g., "*some kids wish they could be a lot better at sports*" and "*other kids feel they are good enough at sports*") and to then indicate whether the selected sentence was "sort of true for me" or "really true for me". The PAC subscale is comprised of six items scored from 0 to 4, yielding a range of possible scores from 6 to 24. Test-retest reliability and internal consistency of the PAC subscale have been shown to be satisfactory, and existing work has supported the hypothesized factor structure of the scale as a whole [24,25]. The PAC subscale was included in the models as a continuous variable. To aid in the interpretation of results, values one standard deviation above and below the observed mean were used to calculate expected levels of enjoyment at "high" and "low" levels of PAC.

#### Individual and demographic characteristics

We selected several covariates found to be associated with enjoyment of PE in children in existing work [9]. The child's height was measured without footwear using a SECA™ portable stadiometer. Weight was measured and recorded to the nearest 0.1 kg using a Tanita™ electronic medical scale. Measured height and weight were used to calculate body mass index (BMI; kg/m^2^) for each participant.

Participation in organized activities was derived from a 63-item measure that assesses past-year participation in free and active play called the Participation Questionnaire (PQ) [23]. Six items provide an inventory of participation in organized athletic and recreational pursuits and sport and dance lessons. The PQ has consistently demonstrated strong construct validity and significant correlations with body fat, aerobic capacity, motoric competence, and other health outcomes [23,25]. Criterion validity has been established using correlations with teacher evaluations of activity (*r *= 0.62); and test-retest reliability of the PQ in a sample of school children in grades 4-6 was measured as 0.81 [23].

Parental education was assessed using a brief questionnaire administered at study baseline. Approximately 63% (n = 1496) of respondents provided information on education. However, as this survey was conducted before the beginning of formal data collection on children, subsequent changes in the composition of the sample and difficulties in matching records meant that this measure was available for only 45.7% (n = 982) of the final sample. We elected to retain the measure in the analysis, including an indicator variable signifying missing values (n = 1168; 54.3%).

### Statistical analysis

We used mixed effects modeling to measure change within individuals over time while adjusting for correlation of measures within children (repeated measures) and within schools. We included random intercepts at the school and student levels, as well as a random slope for time. Analysis of the data revealed possible seasonal effects, so we chose to use an unstructured covariance matrix. We specified three models. The first examines the effects of gender and time on perceived enjoyment of PE, adjusting for baseline age. To identify potential differences between boys and girls in change in reported enjoyment of PE over time, we included a time by gender interaction (Model 1). Next, we added PAC scores, as well as other covariates known to be associated with enjoyment of PE (Model 2). In the final model, we tested for a moderating effect of gender by adding three interaction terms: time by PAC, gender by PAC, and gender by time by PAC (Model 3). This procedure allows for the possibility that the effect of PAC may not be the same between genders across time. All analyses were conducted using SAS version 9.1.

## Results

**Figure 1:**
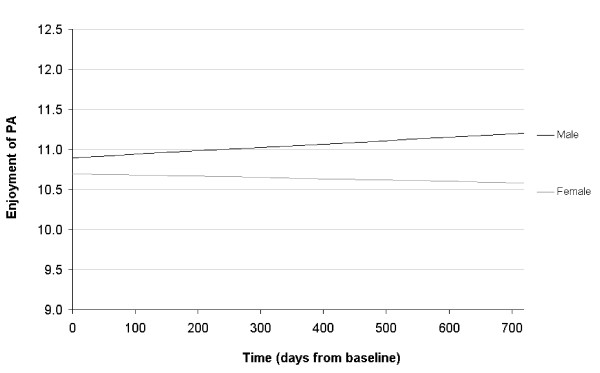
**Enjoyment of physical activity (PA) over time by gender**.

The average age of children at baseline was 9.6 years (SD = 0.34). Slightly more than half of the students were male (50.9%). Table 1 contains descriptive data for enjoyment of PE and all other time-varying measures included in the analysis. Model 1 (see Table 2) shows the effect of time and gender on reported enjoyment of PE. The presence of a significant effect for gender by time (b = 6.50 × 10^-4^, p < 0.001) indicates that there is a divergence of boys' and girls' enjoyment scores. In order to facilitate the interpretation of this result, we have graphed the association between gender, time, and PE using the equation from model 1, illustrated in Figure 1. The Y-axis of this chart, representing predicted values of enjoyment, shows the range from 1 SD below to 1 SD above the total mean. For boys, there is a slight increase (0.18 SDs) in reported enjoyment from grade 4 to grade 6. For girls, however, reported enjoyment of PE slightly decreased (0.08 SDs) over that same period.

**Table 2:** Mixed effects models of the trend in enjoyment of PE over time by gender, perceived competence, parental education, participation in organized activity, and BMI

	Model 1 Estimate (SE)	Model 2 Estimate (SE)	Model 3 Estimate (SE)
Intercept	10.7 (0.05)***	8.41 (0.15)***	9.02 (0.2)***
Time (days)	-1.6 × 10-4 (8.5 × 10-5)	-2.5 × 10-4 (8.2 × 10-5)**	-3.0 × 10-3 (3.5 × 10-4)***
Age (baseline)	0.03 (0.08)	0.03 (0.06)	0.04 (0.06)
Gender			
Male	0.2 (0.07)**	0.01 (0.06)	-0.05 (0.25)
Female	-	-	-
Male by Time	5.8 × 10-4 (1.2 × 10-4)***	5.7 × 10-4 (1.1 × 10-4)***	2.7 × 10-3 (5.4 × 10-4)***
BMI		-0.03 (0.01)***	-0.03 (0.01)***
Parental Education			
High School or Less		-0.16 (0.09)	-0.16 (0.09)
College/Technical School		-0.001 (0.06)	-5.0 × 10-5 (5.5 × 10-2)
Undergraduate Degree		-0.23 (0.09)**	-0.22 (0.09)**
Professional/Graduate Degree		-0.09 (0.11)	-0.08 (0.1)
Education missing		-	-
Organized Activity Participation		1.5 × 10-2 (3.6 × 10-3)***	1.3 × 10-2 (3.5 × 10-3)***
Perceived Athletic Competence (PAC)		0.15 (4.6 × 10-3)***	0.12 (0.01)***
PAC by Time			1.5 × 10-4 (1.9 × 10-5)***
PAC by Male			4.9 × 10-3 (1.3 × 10-2)
PAC by Time by Male			1.2 × 10-4 (2.8 × 10-5)***

** p < .01; *** p < .001

In Model 2, the effect of PAC on reported enjoyment was assessed in concert with other potential covariates. Consistent with the hypothesis, there was a significant effect for PAC, with higher scores associated with greater PE enjoyment, net of the other factors (see Model 2 in Table 2). The coefficient for the interaction between time and gender remained largely unchanged after adjusting for BMI, participation in organized sports, parental education, and PAC. After adjusting for age, gender and time, however, these variables did exert independent effects on enjoyment. Higher BMIs were associated with lower levels of reported PE enjoyment (b = -0.03, p < 0.001), whereas greater participation in sports and organized activities was associated with higher levels of reported enjoyment (b = 0.02, p < 0.001). There was also a weak effect of parental education, with children of parents who hold undergraduate university degrees reporting lower levels of enjoyment of PE than other groups (b = -0.21, p < 0.001).

**Figure 2:**
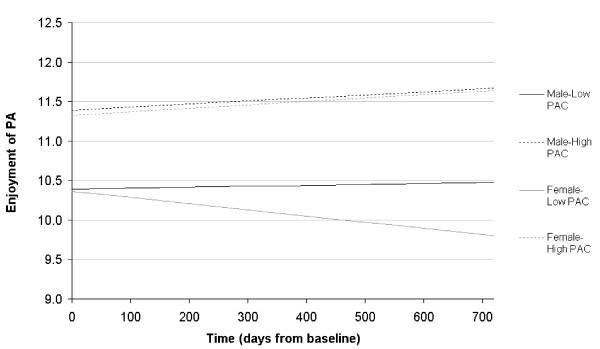
**Enjoyment of physical activity over time by sex and perceived athletic competence (PAC)**.

As shown in Table 2, the introduction of PAC and the other covariates to the equation in Model 2 had very little effect on the coefficient for time by gender (b = 6.30 × 10^-4^, p < 0.001 compared with b = 6.50 × 10^-4^, p < 0.001 from Model 1). This suggests that the effect of PAC on enjoyment over time may differ by gender. In order to test for this, a set of 2-way and 3-way interactions with PAC, time, and gender were added to the model. Complete results from this analysis are shown in Model 3 in Table 2. Most interestingly, a significant 3-way interaction emerged between gender, time, and PAC (b = -1.00 × 10^-4^, p < 0.001). Again, to facilitate the interpretation of this result, we used the full equation in Model 3 to graph the association between PAC, time, and perceived enjoyment for both boys and girls (see Figure 2). In Figure 2, "lower" PAC is represented by a score 1 SD below the mean, and "higher" PAC by a score 1 SD above the mean. The results indicate that gender has no effect on enjoyment of PE for children who perceived themselves as having higher competence with regard to sports and athletics. In fact, over time, there is a slight trend in both boys and girls in this group toward increasing levels of enjoyment. Gender difference did, however, emerge among children with lower perceived competence. Specifically, although children with lower perceived competence persistently had lower levels of enjoyment of PE compared to those with higher perceived competence, the enjoyment levels among boys remained relatively stable over time. Among girls, however, lower perceived competence was marked by decreases in enjoyment of PE over time.

## Discussion

The purpose of the present study was to investigate the association between perceived athletic competence and enjoyment of PE. Drawing on cognitive theories of motivation [24,26], we hypothesized that perceived athletic competence would be positively associated with PE class enjoyment. Generally, the results of this study supported this hypothesis, but the relationship between perceived competence and PE enjoyment was qualified by a 3-way interaction with gender and time. Consistent with previous cross-sectional research [6,15,18-20,26], we found that girls reported lower levels of PE enjoyment than boys. Importantly, however, our results indicate that these differences increased over time. Although previous research has found significant associations between PAC and enjoyment of PE [19], this was the first study to test the association using longitudinal data.

The current findings support previous calls for the targeting of perceived competence in intervention work aimed at increasing PE enjoyment among school-aged children [6,20]. Although the hypothesized PAC and PE enjoyment relationship was supported in this study, it is important to acknowledge that the relationship was complicated by an interaction with gender and time. Specifically, the interaction showed that children of both genders who had higher levels of perceived competence reported higher and increasing levels of PE enjoyment. However, differences emerged among children with lower levels of perceived competence, where boys reported low but stable levels of PE enjoyment over time, while girls began with lower levels of PE enjoyment that continued to decline further over time. These findings offer evidence that gender differences in PE enjoyment are effectively non-existent among children with high levels of perceived competence, and suggest greater efforts should be made to understand why some children have lower perceptions of athletic competence. Improving children's views of their physical abilities may be an important goal, particularly among girls with low perceived competence. The interaction between gender, competence and enjoyment should be a key consideration in planning the content and delivery of PE.

These findings support perceived competence as a focus for interventions designed to increase enjoyment of PE. It is important, however, to understand that competence is just one of many determinants of enjoyment of and participation in physical activity. Cognitive theories of intrinsic motivation, for example, emphasize not only competence, but also perceptions of choice or autonomy [24,27]--constructs that were not measured in this study. A PE program that not only targets positive perceptions of competence, but that also provides a variety of different activities--allowing participants to choose those most of interest to them--is likely to produce greater levels of intrinsic motivation and enjoyment [9,28].

Recent work has also emphasized the importance of understanding sport ability beliefs [29], which underlie feelings of competence because they reflect children's perceptions of the modifiability of their performance [30]. If children perceive their physical abilities to be fixed and not amenable to improvement though practice, it is unlikely that perceptions of enjoyment will be increased in PE settings, where the emphasis is often on raw skill acquisition. One way to improve engagement and perceived competence would be to expose children to a wider spectrum of activities. This would create more opportunities for individuals to discover activities for which they have a particular aptitude or that they find appealing, interesting, and challenging. In fact, this is consistent with the stated policy of PE in the jurisdiction in which this study was conducted. The Ontario Ministry of Education, the governmental body responsible for education curriculums for the schools included in this study, outlines that a major learning goal to be helping students achieve a greater understanding of achieving lifelong health through the promotion of physical and health literacy. Physical literacy, defined as the ability to move, with confidence, across a wide array of physical activities that benefit the whole person (mind and body), being particularly relevant [31]. A caveat, however, was that schools (and teachers) in our study were ultimately responsible for designing and implementing PE classes; which could have varied considerably across school districts depending on available resources (e.g., some schools had state of the art gymnasiums, while other schools did not even have on-site facilities). The idea is to expose children to a greater range of activities, enabling all children to find confidence in different movements suited to individual athletic abilities. Therefore, it was not particularly surprising that higher levels of organized physical activity, as measured by the PQ, were associated with greater reported enjoyment of PE. Overall, focusing on developing competence may not be enough, and understanding and changing negative core beliefs with regard to abilities may also need to be part of the strategy. For example, focusing on individual goal setting, striving for self-set or negotiated standards of performance, and providing greater autonomy and choices in physical activity may be necessary.

Although not a focus of the present study, many of the control variables included in the models were also significantly related to perceived enjoyment of PE. In particular, higher BMI scores were associated with lower level of enjoyment throughout this time period. BMI is of particular interest, as other work has shown that conditions that influence motoric competence are also associated with lower perceived enjoyment of PE [32,33]. Unpacking the temporal associations between these factors and PE enjoyment remains to be done.

The present study has a number of limitations. Although our analysis took correlation within and variation among schools into account with the use of random effects, we were unable to also consider class or school-level factors associated with the delivery of PE (e.g., teacher competence) or the perceived climate in PE class (e.g., supportive, competitive, etc.). While our focus was on individual-level factors, the inclusion of contextual effects would provide a more complete picture; this remains an area for future research. In particular, intervention studies that manipulate teaching environment could shed light on the importance of context in perceived enjoyment.

## Conclusion

Enjoyment of PE makes continued engagement in physical activity more likely; if children do not enjoy PE, they may be less likely to lead an active lifestyle, and to realize the associated health benefits. The results of our study show that girls, in particular, are less likely to enjoy physical education when compared to boys and that this gap in enjoyment grows over time. This may be due to the fact that girls have, on average, lower perceived physical ability. Of course, other factors, not measured in this study (e.g., maturation) may also contribute to observed gender differences. This is the first study to show that the association between perceived enjoyment of PE and PAC is contingent upon gender as well as time. If enjoyment of PE is to be increased, physical educators should attend not only to the development of skills, but also to increasing feelings of competence, particularly competence in PA that the child is able to perceive with relevance to activities inside and outside of the school. In this sense, the mind, as well as the body, must be the focus of instruction in PE class, and should be part of the broader curriculum in physical education for all children; which may be especially the case for young girls.

## Competing interests

The authors declare that they have no competing interests.

## Authors' contributions

JC, JH, and BF are all principle investigators on the PHAST project, and contributed to the design and execution of the study. MYK, SV, and SRB made significant contributions towards the analytic and the intellectual content being presented. All authors read and approved the final manuscript.
